# Robotic Technology in Pediatric Neurorehabilitation. A Pilot Study of Human Factors in an Italian Pediatric Hospital

**DOI:** 10.3390/ijerph17103503

**Published:** 2020-05-17

**Authors:** Francesco Gilardi, Federica De Falco, Daniela Casasanta, Martina Andellini, Simone Gazzellini, Maurizio Petrarca, Andreina Morocutti, Donatella Lettori, Matteo Ritrovato, Enrico Castelli, Massimiliano Raponi, Nicola Magnavita, Salvatore Zaffina

**Affiliations:** 1 Health Directorate, Occupational Medicine Unit, Bambino Gesù Children’s Hospital, IRCCS, 00165 Rome, Italy; francesco.gilardi@opbg.net (F.G.); federica.defalco@opbg.net (F.D.F.); daniela.casasanta@opbg.net (D.C.); 2 Health Technology Assessment Unit, Health Technology & Safety Research Unit, Bambino Gesù Children’s Hospital, 00165 Rome, Italy; martina.andellini@opbg.net (M.A.); matteo.ritrovato@opbg.net (M.R.); 3 Neurorehabilitation Units, Bambino Gesù Children’s Hospital, IRCCS, 00165 Rome, Italy; simone.gazzellini@opbg.net (S.G.); maurizio.petrarca@opbg.net (M.P.); andreina.morocutti@opbg.net (A.M.); donatella.lettori@opbg.net (D.L.); enrico.castelli@opbg.net (E.C.); 4 Health Directorate, Bambino Gesù Children’s Hospital, IRCCS, 00165 Rome, Italy; massimiliano.raponi@opbg.net; 5 Post-Graduate School of Occupational Health, Università Cattolica del Sacro Cuore, 00168 Rome, Italy; nicola.magnavita@unicatt.it; 6 Department of Woman, Child & Public Health, Fondazione Policlinico Universitario A. Gemelli IRCCS, 00168 Rome, Italy

**Keywords:** pediatric neurorehabilitation, robotic therapy, ergonomy, human factor, workload, quality of life, satisfaction

## Abstract

The introduction of robotic neurorehabilitation among the most recent technologies in pediatrics represents a new opportunity to treat pediatric patients. This study aims at evaluating the response of physiotherapists, patients and their parents to this new technology. The study considered the outcomes of technological innovation in physiotherapists (perception of the workload, satisfaction), as well as that in patients and their parents (quality of life, expectations, satisfaction) by comparing the answers to subjective questionnaires of those who made use of the new technology with those who used the traditional therapy. A total of 12 workers, 46 patients and 47 parents were enrolled in the study. Significant differences were recorded in the total workload score of physiotherapists who use the robotic technology compared with the traditional therapy (*p* < 0.001). Patients reported a higher quality of life and satisfaction after the use of the robotic neurorehabilitation therapy. The parents of patients undergoing the robotic therapy have moderately higher expectations and satisfaction than those undergoing the traditional therapy. In this pilot study, the robotic neurorehabilitation technique involved a significant increase in the patients’ and parents’ expectations. As it frequently happens in the introduction of new technologies, physiotherapists perceived a greater workload. Further studies are needed to verify the results achieved.

## 1. Introduction

The application of technological innovation in the health field has brought about relevant benefits for populations’ overall health [1,2]. The introduction of particularly innovative technologies can determine a significant impact on the quality of services offered and health expenditure, thus affecting the health systems as also for the clinical, organizational and economic aspects [3,4].

Among the most recently introduced technologies, the use of robotics technology in upper and lower limb rehabilitation represents an important opportunity to help people affected by different pathologies, e.g., cerebral palsy, diplegia, hemiparesis, paraplegia and others [5,6,7,8]. This perspective opens two aspects that need to be investigated: the effectiveness of integrated care pathways [9] and the role of patients’ engagement in their health care process [10]. Patients’ engagement is of overwhelming importance in pediatric rehabilitation [11]. Parents’ attitude can also be relevant in the complex series of factors that favor the therapy success [12,13]. Robotic therapy requires highly repetitive tasks that can provide substantial improvement when patients remain cognitively engaged in the clinical procedure [7]. Robot-aided neurorehabilitation devices have an approach somewhat similar to a video game, which is appealing to young patients. Previous studies showed that robotic gaming can increase children’s involvement in therapy [14]. This could increase patients’ engagement; electroencephalography studies showed that children remained engaged in the exercises with robotic assistance [15]. Robotics seem capable to activate brain plasticity at the basis of the functional recovery [16], thanks to their interactivity characteristics and intensity of the training experience [17,18,19,20,21,22,23]. 

Healthcare robotics is an emerging area that needs to be studied in depth and supported, so that robotic systems can be positively integrated into the life of patients of all ages and in the physiotherapists’ working activity, deepening their knowledge of the ergonomic aspects that underlie the man–machine interaction. Recently, new high-technology devices have become available for the evaluation, rehabilitation and replacement of lost and non-amendable functions’ purposes [24,25]. The latter is the case of wearable robotic exoskeletons for resuming walking activity that are being experimented in particular with adults with spinal cord paraplegia [26,27]. 

It is important to evaluate what the patients’ expectations are towards this new technology and to compare the level of satisfaction and quality of life they derive from the new treatment, with that in respect to traditional therapies. Patients’ positive predisposition is in fact essential for the success of the treatment [28]. Parents’ attitude, which could affect that of young patients, should also be evaluated for the same reason [29].

Evaluating the occupational impact of the innovation is also essential. It could be hypothesized that, once the first start-up phase of the new techniques—where the physiotherapist’s commitment is probably higher for the adaptation effort—has been overcome, the integration of robotic therapy with the traditional neuro-motor rehabilitation systems may allow a reduction of each operator workload and increase safety when performing high-risk tasks, thus reducing the occurrence of errors and, therefore, increasing the therapeutic efficacy [30]. 

Especially in the first introduction phase of new technologies, a careful attention should be paid to the working conditions and, in particular, the workload that physiotherapists can support in their daily activity. The workload concept has long been recognized as an important individual performance factor within complex systems [31,32,33,34]. The need to evaluate the load imposed on the physiotherapist is particularly critical in high-technology systems [33]. Veltman and Gaillard [35] stated that the measurement of the workload needs behavioral, subjective and physiological data for a complete comprehension of the phenomenon. More recent studies [36] indicate how individual factors are relevant in determining the perception of the workload that should therefore be evaluated in the operator each time a new technology is introduced, in order to avoid abnormal responses that could compromise the results and, therefore, the patients’ care.

Although in the literature studies, evaluating the ergonomic aspects of rehabilitation health technologies is progressively increasing [37], only a few are those are related to robotic devices for neurorehabilitation that also take into account both the physiotherapist and the patient [38,39]. Even rarer are in-depth analyses of these aspects in the pediatric field [40,41,42,43,44,45].

With the perspective of assessing and managing the risks within a pediatric hospital [46,47] associated with the use of robotic devices, it seems necessary to make an assessment that also takes into account the human factor in relation to the characteristics of cognitive ergonomics, usability, software and hardware interface, context and how to use the robotic technology.

Our pilot study, carried out within a health technology assessment (HTA) project funded by the Italian Ministry of Health, has set the following as specific objectives: physiotherapists’ workload evaluation and satisfaction; patients’ quality of life before and after the therapy and their satisfaction; and the expectations and appreciation of patients’ parents regarding the robotic therapy compared with the traditional one.

## 2. Materials and Methods 

### 2.1. Study Design Setting

A pre-post intervention observational study has been developed with the aim of investigating the different aspects of usability and the human factor of the robotic technological therapy compared with the traditional one. The study consists of three surveys relative to (a) the physiotherapists’ workload and satisfaction in using both therapies; (b) the patients’ quality of life and satisfaction before and after both therapies; and (c) parents’ expectations and satisfaction with both therapies. It was an observational study, with the administration at the beginning or during or at the end of the treatment of the self-completed questionnaires.

The study was carried out at the neurorehabilitation department of an Italian pediatric hospital.

### 2.2. Traditional and Robotic Technology

Traditional rehabilitation therapy mainly consists of functional and goal-oriented training, whose efficacy is well reported in the literature [25]. It is realized with exercises tailored to the specific motor abilities of the patients. 

The Lokomat^®^ System (Hocoma Inc., Volketswil, Switzerland) is the most diffuse and exhaustive technology for robotic-assisted gait recovery. It consists of the following: (a) a bilateral “driven gait orthosis (DGO)” system; (b) a body weight support for each patient through the Levi system; (c) a treadmill; and (d) a virtual reality including knowledge of both the results and performance. All these modules were employed during the training. The purpose of the device is to deliver a series of steps cycles to the patient’s lower limbs, performing a control of the same limbs on the sagittal plan [48]. Robot-assisted gait training is a promising technique to restore functional walking and improve locomotor ability, which might enable patients to maintain a healthy lifestyle and increase their level of physical activity [49]. 

Lokomat is a very common robotic therapy in rehabilitation centers. The experiences conducted on the various neurological pathologies are analyzed through an international network [50]. Therapeutic applications of exoskeleton-based rehabilitation methods, such as Lokomat, have been compared with end effector robot (G-EO) training or manual-assisted partial-body weight-supported treadmill training (PBWSTT) in clinical samples [51,52,53].

The specific abilities of the patients determine also the neurologist’s decision about the administration of only Lokomat or a mixed approach. In particular, patients unable to walk autonomously received only Lokomat, while the others underwent mixed interventions. Each session of treatment consisted of 45 min of therapy, for each treatment. The patients received daily sessions for 5 days a week. All the operators were physiotherapists with long-term experience in teamwork.

### 2.3. Enrollment of Study Populations Inclusion and Exclusion Criteria

A sequential patient enrollment was carried out in the neurorehabilitation unit of a pediatric hospital. All patients who performed a cycle of therapy within the study period (January–December 2019) with the robotic, traditional or mixed technology (traditional and with Lokomat neurorehabilitation robotic technology) were invited to participate. Enrollment also involved each patient’s parents. 

The inclusion criteria were:

1. Patients
  - Children aged over 4 and under 16 years;
  - Children affected by a neurological pathology that required neurorehabilitation therapy of the lower limbs;
  - Hospitalized for a neurorehabilitation therapy cycle with the use of the Lokomat robotic technology, exclusive or mixed with traditional therapy, or an exclusively traditional therapy;
  - Presenting cognitive medium/low-degree deficits;
2. Parents
  - Father and mother of each child selected for the study;
3. Physiotherapists
  - Trained in the use of the technology;
  - Usually engaged in the therapy that uses the technology;
  - Usually engaged in the traditional therapy.

The exclusion criteria for the patients were the presence of a high-degree cognitive deficit that did not allow the questionnaires’ administration. Patients with a medium-high cognitive impairment, who in any case presented a sufficient capacity for completing the questionnaires, were included in the study.

The number of eligible patients in the study period was 48 units; 46 of them, equal to 95.8%, joined the study.

The pediatric patients enrolled in the study were 46 (24 males, 52.2%) with an average age of 9.7 years (SD ± 3.80). The patients’ main characteristics inherent in the main socio-demographic variables are reported in Table 1.

The parents of pediatric patients enrolled in the study were 47 (40 females, 85.1%) with an average age of 43.4 (SD ± 8.14). According to the type of treatment to which the child was subjected, they were classified in the robotic group (*n* = 16), traditional group (*n* = 15) or mixed group (n = 16). 

The physiotherapists working for the hospital neurorehabilitation unit who carry out the therapeutic sessions were a total of 12 (8 females, 66.7%), of which 4 were operating for both therapies and 8 only for the traditional therapy. The physiotherapists’ average age was 38.8 years (SD ± 12.14); their working experience was on average 14 years, with about 4 years of experience in the use of the Lokomat robotic technology for those specialized in its use.

### 2.4. Study Variables 

For the study purposes, several socio-personal variables were taken into consideration: (a) physiotherapists’ gender, age, length of service, experience in the use of the technology; (b) patients’ gender, age, pathology, clinical evaluations; and (c) parents’ gender, age, educational qualification, employment, marital status, number of children (Table 2).

The variables related to the various ergonomic dimensions were also measured, through the use of validated questionnaires.

For the physiotherapists, the following was assessed: workload evaluated through the NASA Task Load Index questionnaire (NASA-TLX), which was administered at the end of each therapy for the entire length of the study—12 months; and job satisfaction was evaluated through the Job Satisfaction Scale of Warr et al. (single administration at the beginning of the study).

For the patients, quality of life was measured at the beginning and at the end of therapy, through the quality of life questionnaire, pediatric version (Pediatric Quality of Life Inventory ^TM^) Measurement Model, (PedsQL TM 4.0 SF15 Generic Core Scales), as well as satisfaction at the end of therapy (Net Promoter Score (NPS) questionnaire).

For parents, expectations regarding the therapy (ad hoc questionnaire administered at the beginning of therapy) and satisfaction with the therapy (NPS questionnaire administered at the end of therapy) were assessed. A synthetic description of the type of variables and their nature is summarized in Table 2. 

Comparisons were made on the two therapies: robotic and traditional. Patients’ quality of life referred to the type of therapy (robotics, traditional or mixed) followed by each of them. 

### 2.5. Questionnaires 

The workload questionnaire, NASA-TLX [54,55], is a subjective, multidimensional assessment tool that rates the perceived workload in order to evaluate the performance. It provides a general workload score based on the weighted average of six sub-scales: psychological demands, physical demands, temporal demands, performance, effort and frustration. The criteria for the composition of the subscales and their relative weight on the overall workload are detailed in the NASA manual [56]. Initially, the meaning of each of these dimensions is explained to the subject who is asked to evaluate in which position, on a twenty-point scale, the performed task is located. High scores indicate heavy requests and low scores the negligible ones. Subsequently, all possible pairs of scales are presented to the subject, who has to indicate for each pair which dimension is most relevant in determining the workload. Based on these comparisons, the authors determined some weights which are then used to calculate, starting from the initial evaluations, a general workload score ranging from 0 to 100. 

The Warr’s Job Satisfaction Scale [57,58] is a questionnaire assessing job satisfaction. It is a scale composed of 15 questions, plus a 16th one that asks what level of satisfaction derives from all the factors covered in the previous questions. Each question is answered on a seven-point Likert scale, from “extremely dissatisfied” (=1) to “extremely satisfied” (=7). The total score of the 15 questions ranges from 15 to 105. The authors recommend to distinguish the intrinsic professional satisfaction, given by the seven even questions, from the extrinsic satisfaction, corresponding to the seven odd questions.

The PedsQL (Pediatric Quality of Life Inventory) Measurement Model is a modular approach to measure health-related quality of life in healthy children and adolescents and those with acute and chronic health conditions [59,60,61]. We used the questionnaire PedsQL TM 4.0 SF15 Generic Core Scales for children between 4 and 16 years of age. It is made up of 23 items spread over 4 areas of pediatric health suggested by WHO: physical, emotional, social and scholastic. Subjects have to state their degree of agreement on a five-point Likert scale from 0 (never) to 4 (almost always). The score obtained is converted into a scale from 0 to 100, where the highest score represents the highest level of functioning. The reference scores of the questionnaire are the physical health summary score (PhyHSS), psychological health summary score (PsyHSS) and total score (TS).

The Net Promoter Score (NPS) [62,63] is a measure of customer experience. It measures the proportion of “promoters” of a product, compared to its “detractors”. The number can range from −100 (all are detractors) to +100 (all are promoters). NPS is based on a single question to be asked to those who have used the product: “How likely you are to recommend this product to a friend or colleague”? Parental satisfaction, measured through the NPS questionnaire, was investigated with reference to six dimensions: therapeutic path, operator, quality of the information received, quality of assistance, technological innovation and achievement of goals. The level of parental expectations was measured by means of a questionnaire developed ad hoc and expressed as the difference between the two therapies—robotic and traditional—as two questionnaires that were administered to the parents of those patients undergoing mixed therapy, one for each of the two therapies.

The questionnaires were self-completed after a suitable explanation by trained personnel and offered to the patient, parents and physiotherapists during the time provided for by the protocol in the expected times with respect to the cycle of therapy. A complete description of the population, dimension explored, type of questionnaire and timing of administration is summarized in Table 3.

### 2.6. Statistical Analysis

The statistical analysis was first carried out on a descriptive basis, defining as the mean the score standard deviation of each questionnaire. Frequencies and percentages were calculated for the qualitative variables. 

Since the patients underwent robotic therapy or traditional therapy, or both, we were able to compare the three groups with reference to their quality of life. All the other questionnaires were administered only with reference to the comparison between robotics and traditional therapy.

For comparisons of means between the two groups, a Student unpaired t-test was used for the normally distributed variables, whereas the Mann–Whitney–Wilcoxon U test has been used for the non-normally distributed variables. A non-parametric analysis of variance using the Friedman test was used for the comparison among the three groups (Lokomat therapy, traditional therapy, mixed therapy). All statistical analyses were performed using the SPSS Statistics software (IBM SPSS Statistics V25, Chicago, IL, USA).

### 2.7. Ethical Considerations. Informed Consent

The study conforms to the ethical principles of the Good Clinical Practice, the Helsinki Declaration, and is in compliance with the current regulations. The Independent Ethics Committee of Bambino Gesù Children’s Hospital (Protocol n. 1787/2019) gave its approval. The informed consent was obtained when the patients were enrolled in the study. 

## 3. Results

The comparison of the workload self-assessment scores with the NASA-TLX questionnaire indicates that the therapist involved in the use of the robotic technology perceives a significant higher overall (total score) workload compared with the colleagues who use only the traditional techniques (Table 4). More specifically, observing the trend of the subscales of the NASA-TLX, the performance is considered much better by those who are working with Lokomat than the others, and the physical and temporal load are substantially superimposable. On the contrary, while the therapists’ performance, under a statistical point of view, resulted as significantly higher in those who perform the traditional therapy compared with the robotic one, efforts and frustration are higher in those who are struggling with the new technique than with physiotherapists who apply the usual manual techniques.

Considering the Job Satisfaction Scale, physiotherapists who use the Lokomat therapy present higher scores compared with the colleagues who work only with the traditional techniques, both in the total score and in the intrinsic/extrinsic professional satisfaction, even if the difference does not reach a statistical significance.

Results from the PedsQL questionnaire revealed that patients who have undergone the Lokomat robotic therapy significantly increased their quality of life (PhyHSS; PsyHSS and TS) when comparing the pre- and post-therapy scores. Patients who have undergone mixed therapy, as well as the traditional one, showed a statistically significant increase in the quality of life (PhyHSS and PsyHSS scores) when comparing the pre- and post-treatment answers, whereas no significant differences were found in the total scores. The results of the pre- and post-quality of life tests are reported in Table 5.

Patients’ satisfaction, measured by the NPS questionnaire, showed high values (above or around 60%) for the two types of therapy (LOKOMAT, traditional) with a higher satisfaction for the Lokomat therapy, which recorded a score of 65.4%, compared with 57.2% of the traditional therapy.

Parents’ expectations regarding the Lokomat robotic therapy are moderately higher than the traditional therapy, however, are not statistically significant.

**Figure 1:**
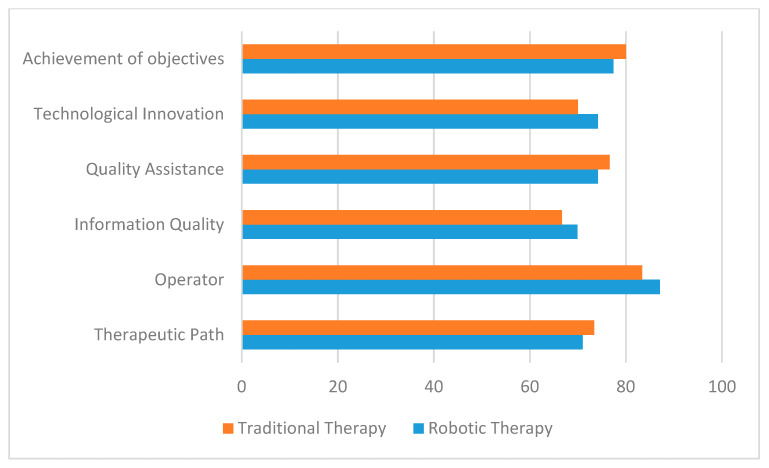
Degree of satisfaction expressed by parents: comparison between traditional and robotic therapy.

Parental satisfaction expressed at the end of the therapeutic path is positive (above 65%) on all dimensions investigated for both therapies even though no significant differences emerged. The robotic therapy results were also associated with a higher satisfaction level than the traditional one with respect to the “operator”, “quality of information” and “technological innovation” dimensions (Figure 1).

## 4. Discussion

This study has allowed us to demonstrate that robotic neurorehabilitation therapy is welcome by patients and parents, and that physiotherapists get some professional satisfaction from it even if in the first stage of its application, physiotherapists experience an increased effort. The augmented perception of workload in those who have to apply a new technique (which was expected) is not so high and therefore does not raise concerns for physiotherapists’ and patient’s safety. This increase is mainly due to the increased psychological demand and effort required by learning a new technique, and to the foreseeable frustrations that derive from this effort. However, personal performance is considered much better by those who work with Lokomat than by others who do not. Moreover, generally, physiotherapists who use Lokomat besides the traditional therapies are more satisfied with their work than their colleagues. This should mean that the new technology will enter the pediatric neurorehabilitation heritage without problems.

These observations meet what was reported in the literature, according to which ergonomic aspects in physiotherapists are among the priorities to be observed [64], even if there is still a lack of systematic approaches in the ergonomics risks assessment [65] and a common failure to apply the principles of ergonomics in pediatric rehabilitation in daily practice [66]. Human factors and physiotherapists’ expertise are of mainstream importance in neurorehabilitation practice [67]. The ergonomic well-being of physiotherapists, their satisfaction and motivation are important for them to carry out their supervisory tasks correctly, hence improving the quality of care for young patients [68].

Quality of life values show a clear improvement in the group of patients submitted to robotic therapy. The satisfaction expressed by parents, patients and physiotherapists shows overall higher results for robotic therapy compared with traditional therapy.

These results confirm and widen the observations reported in the literature. The majority of the topic-related published studies were conducted on a few patients, especially adults and those affected by pathologies, such as stroke and spinal cord paraplegia. In order to evaluate the usability and acceptability of robotic rehabilitation systems, the authors used self-filled questionnaires, such as the System Usability Scale [69] and the Use Questionnaire [70]. Three studies qualitatively assessed the expectations and experiences of pediatric patients affected by cerebral palsy and families regarding the use of Lokomat [41,42,43], and gathered positive results about the acceptability of the therapy. One study reported that the technology, as delivered in an in-patient setting, may have a positive psychosocial impact [40]. According to a study carried out in Colombia, most of the patients that use Lokomat as a rehabilitation therapy feel comfortable (47%), very safe (68%) and have a perspective of significant results with the therapy (68%). However, when comparing the number of patients in therapy with Lokomat with the number of people that have gait disabilities, it results that only few Colombians have access to this type of therapy [71]. Therefore, the real problem for public health is not the introduction of robotic therapy, but its limited accessibility.

Only one published study analyzed together the expectations of pediatric patients and families, focused on the physiotherapists’ expectations and the families’ involvement in the therapeutic process. The results show the importance of using the right strategies to preserve the families’ expectations by maintaining relationships of trust, simultaneously guaranteeing the evidence-based treatment plan and identifying achievable goals [44].

This study has some limitations and some strengths. First of all, the limitations are due to the fact that it was conducted only in one hospital, and on a limited number of patients and physiotherapists. We could not randomize the observations or treatments. Only the collection of a higher number of workers and patients would allow to verify the importance of all the possible confounding factors. However, the purpose of this study was not to evaluate the efficacy of robotic therapies compared with the traditional ones in the various childhood diseases, but only to assess the impact that the introduction of the new techniques in a small rehabilitation center had on patients, parents and physiotherapists. In fact, this is a pilot study, whose results have to be taken with great caution and should be verified on a larger number of cases. However, the study presents several aspects of originality and innovation. The research constitutes, indeed, a first study specifically dedicated to the ergonomic and behavioral aspects connected to the use of robotic technology in pediatric neurorehabilitation, which simultaneously takes into account the point of view of patients, parents and physiotherapists. Further studies might investigate these and other ergonomic aspects by relating them to the actual clinical efficacy of the treatment.

## 5. Conclusions

This pilot study highlighted the positive impact of robotic neurorehabilitation therapy on patients, their families and physiotherapists, detectable by the expectations and satisfaction level indicated in the various evaluations performed and during the relationships between physiotherapists, patients and family members with the researchers during the various phases of the project. Further studies, conducted on a larger number of patients and physiotherapists will confirm whether robotic techniques allow a greater “engagement” of patients and caregivers, as some authors have postulated.

It is recommended to carry out further investigations, such as the one proposed in this study, in all the services that introduced these new technologies, as they can confirm the importance of the bio-psycho-social framework when dealing with patients under intensive neurorehabilitation for motor impairments with assistive devices. Considering the world being in continuous evolution, both in everyday life expectations and more specifically in high-technology rehabilitation, further studies are needed to focus on the bio-psychosocial impact of robot-assisted rehabilitation.

A continuous research is strongly motivated by the attention, expectation and satisfaction of the users and the aspects assessable through an analysis of the therapy’s ergonomic aspects, that constitutes a significant aspect of the clinical effectiveness of the robotic therapy. It also highlights and strengthens, through a careful and constant feedback from the users, the real value and the actual impact of the man–machine interaction in the therapeutic setting. In fact, utility does not correspond with effectiveness, but it is strictly connected to multiple factors including usability, consideration of expectations and satisfaction, with an enhancement of the physiotherapist/patient/family relationship.

In this context, and even more in the introduction and application of the technology to the rehabilitation process, the physiotherapist plays a central role of the mediator in the use of the robotic therapy, which requires an in-depth investigation to understand the possible functioning, mechanisms and, above all, the effects on training and updating.

## Figures and Tables

**Table 1:** Characteristics of the patients enrolled in the study.

		Patients	Lokomat	Mixed	Traditional
Number		46	15	15	16
Age		9.65 (DS ± 3.80)	10.20 (DS ± 4.30)	9.20 (DS ± 3.63)	9.56 (DS ± 3.54)
Sex	Male	24 (52.2%)	9 (60%)	8 (53.3%)	7 (43.7)
	Female	22 (47.8%)	6 (40%)	7 (46.7%)	9 (56.3%)
Diagnosis	Double hemiparesis	14 (30.4%)	6 (40%)	7 (46.7%)	
	Diplegia	9 (19.6%)	4 (26.7%)	3 (20%)	2 (12.5%)
	Inf. cerebral palsy	4 (8.7%)	1 (6.6%)	2 (13.3%)	2 (12.5%)
	Hemiparesis	4 (8.7%)	-	1 (6.7%)	2 (12.5%)
	Other	15 (32.6%)	4 (26.7%)	2 (13.3%)	10 (62.5%)
Cognitive Level	Normal	23 (50%)	7 (46.7%)	10 (66.6%)	12 (75%)
	Slight impairment	10 (21.7%)	4 (26.7%)	3 (20%)	3 (18.7%)
	Medium impairment	5 (10.9%)	3 (20%)	1 (6.7%)	1 (6.3%)
	Medium-severe impairment	2 (4.3%)	1 (6.6%)	1 (6.7%)	
Average Length of Treatment (days)		16.8 (DS ± 8.23)	16.6 (DS ± 8.10)	19.9 (DS ± 8.27)	14.1 (DS ± 7.84)

**Table 2:** Socio-demographic variables considered in the study.

Variable	Description	Type of Measure
Physiotherapists		
Gender	Male/Female	Categorical
Age	Years	Continuous
Length of service	Years	Continuous
Experience in the use of the technology	Years	Continuous
Patients		
Gender	Male/Female	Categorical
Age	Years	Continuous
Pathology	Diplegia; Hemiparesis; Cerebral palsy; Others	Categorical
Cognitive level	None or Low; Medium; Severe Impairment	Categorical
Parents		
Gender	Male/Female	Categorical
Age	Years	Continuous
Education	<8; 8–13; >13 years of schooling	Categorical
Employment	Housewife of housemaker; white collar; blue collar	Categorical
Marital status	Single; Paired	Categorical
Children	Number	Discrete

**Table 3:** Framework of ergonomic dimensions explored, questionnaires and time of administration.

Category	Questionnaire	Measure	Time of Administration/Schedule
Patients	PedsQL	Quality of life	Pre and post the cycle of therapy
	NPS	Satisfaction	At the end of the cycle of therapy
Parents	Ad-hoc	Expectations	At the beginning of the cycle of therapy
	NPS	Satisfaction	At the end of the cycle of therapy
Physiotherapists	NASA-TLX	Workload	At the end of each therapy
	NPS	Satisfaction	At the end of the cycle of therapy

**Table 4:** Physiotherapists’ workload (NASA-TLX questionnaire).

Subscale	Lokomat		Traditional		*p*-Value *
	Mean	SD	Mean	SD	
Psychological Demands	23.87	±7.06	21.05	±8.07	0.062
Physical Demands	14.21	±9.06	14.58	±9.87	0.098
Temporal Demands	0.90	±1.71	0.99	±1.57	0.711
Effort	20.85	±6.73	18.18	±6.74	0.047
Frustration	9.85	±9.84	2.34	±2.91	<0.001
Total Score	83.56	±9.32	77.17	±8.76	<0.001

* Mann–Whitney U Test.

**Table 5:** Quality of life scoring measured before and after the therapy cycle (PedsQL questionnaire for the various age groups).

Score		Lokomat			Mixed			Traditional			*p*-Value **
		Mean	SD	*p*-Value *	Mean	SD	*p*-Value *	Mean	SD	*p*-Value *	
Psychological Health Summary Score (Emotional, Social and School Functioning Scale Score)	PRE-TEST	73.7	±13.31	0.004	71.0	±15.55	0.05	72.5	±13.81	0.04	0.62
	POST-TEST	78.1	±12.38		75.6	±11.43		75.4	±14.55		
Physical Health Summary Score	PRE-TEST	38.8	±14.65	0.01	40.6	±13.16	0.005	43.4	±21.28	0.01	0.35
	POST-TEST	50.2	±15.86		49.6	±15.47		51.3	±17.31		
Total Score	PRE-TEST	60.9	±11.40	0.04	60.4	±11.73	0.08	62.2	±12.13	0.37	0.55
	POST-TEST	66.10	±13.48		65.0	±9.96		62.9	±13.05		

* Wilcoxon Test; ** Friedman Test.

## Data Availability

The datasets used and/or analyzed during the current study are available from the corresponding author on reasonable request.
